# Chile’s role in global health diplomacy: a narrative literature review

**DOI:** 10.1186/s12992-018-0428-8

**Published:** 2018-11-16

**Authors:** Jorge Ramírez, Leonel Valdivia, Elena Rivera, Marilia da Silva Santos, Dino Sepúlveda, Ronald Labonté, Arne Ruckert

**Affiliations:** 1 Program of Global Health, School of Public Health, Faculty of Medicine, Universidad de Chile, Santiago de Chile, Avenida Independencia 939, Independencia, Santiago, Región Metropolitana Chile; 2 Globalization and Health Equity Research Unit, School of Epidemiology and Public Health, University of Ottawa, 600 Peter Morand Crescent, Ottawa, ON K1G 5Z3 Canada

**Keywords:** Global health diplomacy, International relations, South-south cooperation, Foreign policy, International health, Public health

## Abstract

**Background:**

Global health diplomacy (GHD) has become an important field of investigation due to health concerns increasingly entering the foreign policy domain. Much of the existing academic writing focuses on North-South cooperation in global health, and emphasizes the role of security and economic interests by Northern countries as drivers of GHD. Chile presents a favourable environment for an expanded involvement in future GHD activities. However, there is little knowledge about what has been driving Chile’s integration of health into foreign policy, and little effort to appropriate knowledge from international relations theories to better theoretically grasp the emergence of GHD.

**Methods:**

To fill this knowledge gap, we conducted a narrative literature review of the driving forces behind Chile’s integration of health into foreign policy. Drawing on a popular analytical framework used in international relations scholarship, we identified driving forces of the integration of health into Chile foreign policy at three levels of analysis.

**Results:**

At the international/global level of analysis, the main driving forces were related to national security concerns and compliance with regulations of international organizations. At the regional level, GHD was driven by a commitment to regional solidarity through mutually beneficial cooperation in response to neoliberal reforms; health coordination in emergencies; and protection of indigenous peoples. Finally, at the domestic level, drivers identified include economic interests of various productive sectors and how health regulations might impact those; the high degree of social inequity which impacts on access to healthcare; and management of natural disasters.

**Conclusion:**

Health actions in the context of international relations in Chile are still mainly motivated by more traditional foreign policy interests rather than by a desire to satisfy health needs per se. This seems to conform with findings of existing GHD scholarship that emphasize the importance of security and economic interests as driving forces of GHD, and how health is often appropriated instrumentally within foreign policy settings to achieve other goals. But the review also reveals that in the context of South-South cooperation (and regional health diplomacy), solidarity and normative considerations can be important driving forces as well. Finally, the review demonstrates that there has been an evolution from chiefly domestically focused health policies (e.g. maternal and child nutrition treatment) towards internationally inspired integrated policies (e.g. maternal and child nutrition promotion aligned with international guidelines).

**Electronic supplementary material:**

The online version of this article (10.1186/s12992-018-0428-8) contains supplementary material, which is available to authorized users.

## Resumen

**Introducción:**

La diplomacia en salud global (DSG) se ha convertido en un importante campo de investigación debido a que los problemas de salud son cada vez más considerados en el ámbito de la política exterior. Gran parte de la literatura existente se centra en la cooperación Norte-Sur en salud global, y enfatiza el papel de la seguridad e intereses económicos por parte de los países del Norte como impulsores de la DSG. Chile presenta un escenario favorable para una mayor participación en actividades de DSG. Sin embargo, existe poco conocimiento sobre lo que ha estado impulsando la integración de la salud de Chile en la política exterior, y pocos esfuerzos por usar los elementos de las relaciones internacionales y las teorías de la política exterior para entender mejor el surgimiento de DSG.

**Método:**

Para llenar este vacío de conocimiento, realizamos una revisión narrativa de la literatura. Basándonos en un popular marco analítico utilizado en estudios sobre relaciones internacionales, identificamos las fuerzas que impulsan la integración de la salud en la política exterior de Chile en tres niveles de análisis.

**Resultados:**

En el nivel de análisis internacional / mundial, las principales fuerzas motrices se relacionaron con las preocupaciones de seguridad nacional y el cumplimiento de las reglamentaciones de las organizaciones internacionales. A nivel regional, la DSG se ha impulsado por: un compromiso con la solidaridad regional a través de la mutua cooperación en respuesta a las reformas neoliberales; una necesidad de coordinación de salud en emergencias; y la protección de pueblos indígenas. Finalmente, a nivel doméstico, los factores identificados incluyen los intereses económicos de varios sectores productivos (y cómo las reglamentaciones sanitarias podrían afectarlos); el alto grado de inequidad social que impacta en el acceso a la atención médica; y el manejo de desastres naturales.

**Conclusiones:**

Las acciones de salud en el contexto de las relaciones internacionales en Chile siguen estando motivadas principalmente por intereses de política exterior tradicionales, más que por el deseo de satisfacer las necesidades de salud per se. Esto parece concordar con hallazgos previos, que enfatizan la importancia de la seguridad y los intereses económicos como fuerzas impulsoras de la DSG, y cómo el tema de salud suele ser apropiado instrumentalmente dentro de la política exterior para lograr otros objetivos. Sin embargo, la revisión también revela que, en el contexto de la cooperación Sur-Sur (y la diplomacia regional de salud), la solidaridad y las consideraciones normativas también pueden ser importantes fuerzas impulsoras. Finalmente, ha existido una evolución de las políticas de salud centradas principalmente en el ámbito interno (p. Ej, el tratamiento de nutrición materno-infantil) hacia políticas integradas inspiradas a nivel internacional (p. Ej., la promoción de la nutrición materno-infantil se alinea con las directrices internacionales).

## Background

Global health diplomacy (GHD) has become an important field of academic investigation due to health concerns increasingly entering foreign policy considerations. Although subject to many interpretive definitions, GHD is most simply defined as the multi-level and multi-actor negotiations that shape the global policy environment for health [1]. This includes various efforts through which States, intergovernmental organizations, and non-State actors negotiate responses to global health challenges (in which improved health is the identified policy goal); but also encompasses using health concepts or mechanisms in global policy negotiations to achieve other political, economic, and social objectives (in which health is a means to other foreign policy objectives such as security) [2]. The merging of health and foreign policy is linked to the securitization of health, with recent outbreaks of various epidemics (SARS in 2003, Influenza H1N1 in 2009, and Ebola in 2014) demonstrating how infectious diseases can spread rapidly across the globe, affecting traditional foreign policy concerns of security, travel and trade [3].

Much of the existing academic work focuses on North-South cooperation in global health, and emphasizes the role of security and economic interests by (historically dominant) Northern countries as drivers of GHD. Less has been written about what is driving Southern countries to partake in GHD, and how to theorize about their role in GHD; a knowledge gap this review is focused on closing [4]. Although in Chile the integration of health into the foreign policy agenda is not immediately visible in official diplomatic channels (i.e. at the Ministry of Foreign Affairs [MINREL] and its network of specialized agencies), our findings reported below demonstrate that Chile has been a vital player and keen promoter of important regional and global health initiatives.

Chile returned to a democratic regime in 1990 after being ruled by a military government for almost 17 years. A center-left political coalition of Christian Democrats, Socialists and other progressive parties (The “Concertación”), controlled the government until 2010 when it was replaced by a right-wing political coalition for 4 years, before reverting back to a socialist president in 2014. Since the early 1990s, the official priority of the Chilean foreign policy agenda has been the promotion and expansion of the commercialization of Chilean products (e.g. copper, timber, fish, fruit and wine, among others) and expansion into global markets. The principal vehicle for achieving this goal has been the establishment of a string of free trade agreements (FTAs) with over one hundred nations and regional associations around the world. At the same time, Chile has experienced a growing involvement with its immediate neighbours and the rest of the Latin American region, with emphasis in regional health initiatives. Examples in our analysis include cross border cooperation in the North of Chile (focusing on intercultural health and security issues); Chile’s active involvement in the Union of South American Nations (UNASUR), the Common Market of the South (MERCOSUR), and the Andean Community of Nations (CAN), each of which has undertaken important health initiatives; and the fast-paced immigration of workers and families from neighbouring nations (including health workers).

These developments constitute a favourable scenario for an expanded involvement of Chile in future GHD activities. However, there is little knowledge about what has been driving Chile’s integration of health into foreign policy, and little effort to appropriate knowledge from international relations (IR) theories to better grasp the emergence of GHD [3], especially so in the context of South-South Collaboration (SSC) [4]. Our first step in filling this knowledge gap was to systematically gather and analyze what existing studies and related commentaries have documented on these two issues. Our findings suggest that there are a number of competing and complex motivations behind the integration of health into Chile’s foreign policy which represent a mix of security, economic, and normative interests, with no single IR theory able to comprehensively capture Chile’s GHD dynamics.

## Methods

We conducted a narrative literature review with the goal to identify: 1) what health concerns enter diplomatic and foreign policy discussions and negotiations in Chile; 2) how they are framed in this process; 3) what are the driving forces for their inclusion into foreign policy; and 4) how we can theoretically understand these processes by drawing on IR theory (the last two of which are the focus of this article). We decided to conduct a narrative review for two reasons; first it allowed us to “identify what has been accomplished previously, allowing for consolidation, for building on previous work, for summation, for avoiding duplication and for identifying omissions or gaps” [5] in a field where a paucity of articles is expected due to its recent development; and second, because our research questions were theoretical and conceptual in nature, a narrative literature review set up (as opposed to a systematic review) allowed us to go beyond mere description of GHD practices to facilitate conceptual engagement with IR theories.

The review is part of a larger comparative research project to better understand GHD practices in Brazil, Canada, Chile, and Mexico and follows the study protocol of that larger research project, with a global literature review of GHD recently published elsewhere [3].

We reviewed seven electronic data bases (PubMed, ASCE, Web of Science, BIREME, INIA, Scielo and EBSCO (see Additional file 1). We collected articles mainly in Spanish but we also included some articles in English whenever we found scholarship on Chilean GHD published in English. Due to the increase in global health diplomacy activities after the turn of the millennium, we considered articles published between 2000 and 2015. The list of search terms can be found in Additional file 2 (translated into English with the actual search performed in Spanish). Most of the articles were published by independent scientific journals and written by scholars in different fields of study. Only 4 articles were found in institutional publications from the government or journals belonging to government institutions.

After removing the duplicates, we screened the articles through abstract reviews, selecting articles that satisfied at least one (or more) of the following inclusion criteria: (i) cover international policy issues relevant to health due to their direct and/or indirect implications; or (ii) discuss (explicitly or implicitly) how health issues are framed/ incorporated in foreign policy negotiations; or (iii) analyze the forces involved in the integration of health in foreign policy negotiations; or (iv) explore the ways in which diplomacy in global health is practiced; or (v) analyze explicitly and/or implicitly the bases or theoretical foundations of the integration of health in international politics, in terms of international relations theory (see Additional file 1 for details).

Searches identified 763 potentially relevant articles, and 582 after removal of duplicates. The review of these titles yielded 78 articles after abstract screening. These articles were read in their entirety by three teams of two reviewers each. If there was disagreement within a team of two relating to selection of an article, a third party was consulted. Finally, 32 of them were selected for further analysis due to their relevance for addressing the portion of review findings that we are reporting in this article (goals 3 and 4 described above) and were coded and reported in this article (see Table 1). Coding was performed utilizing NVivo 10 and a coding tree developed by the Canadian research team, with some modifications considered appropriate to the particularities of the Chilean context (see Additional file 3). We also developed an integrative narrative of the findings in terms of how international relations theory can be used to facilitate a better understanding of the motivating factors behind Chilean GHD.

**Table 1:** Articles selected and used for the analysis

Year	Title (Original)	Journal	Author
2000	La atención gerenciada en América Latina. Transnacionalización del sector salud en el contexto de la reforma	Cadernos De Saúde Pública	Iriart et al.
2001	Managed care in Latin America: the new common sense in health policy reform	Social Science & Medicine	Iriart et al.
2004	El tratado de paz y amistad de 1984, la cooperación militar y los problemas limítrofes entre Chile y Argentina	Revista Universum	Mendoza
2006	Pneumococcal disease and vaccination in the Americas: an agenda for accelerated vaccine introduction	Revista Panamericana De Salud Pública	Garcia et al.
2007	Farmacovigilancia en medicina veterinaria: una perspectiva desde el punto de vista internacional y situación actual en Chile	Archivos De Medicina Veterinaria	Iragiien et al.
2007	Los Determinantes Sociales de Salud y la lucha por la equidad en Salud: desafíos para el Estado y la Sociedad Civil	Revista Saúde E Sociedade	Villar et al.
2007	Reglamento Sanitario Internacional (RSI)	El Vigia	Arredondo et al.
2008	Aproximaciones políticas a la lucha contra la desnutrición infantil en América Latina y el Caribe		United Nations
2008	Food-based dietary guidelines and implementation: lessons from four countries--Chile, Germany, New Zealand and South Africa	Public Health Nutrition	Keller et al.
2009	Antecedentes internacionales y nacionales de la promoción de salud en Chile: Lecciones aprendidas y proyecciones futuras.	Revista Chilena De Nutrición	Crovetto et al.
2009	Cambio Climático Y Salud En La Región Andina	Revista Peruana De Medicina Experimental Y Salud Pública	Feo et al.
2010	El proyecto subregional Cono Sur de control y vigilancia de la Hidatidosis	Revista Peruana De Medicina Experimental Y Salud Pública	Irabedra et al.
2010	Licencias obligatorias por razones de salud pública en Chile. Un análisis comparativo con los acuerdos de la ADPIC	Revista Lus Et Praxis	Cerda et al.
2010	Objetivos de desarrollo del milenio: tercer informe del Gobi…	Gobierno De Chile Y Naciones Unidas	Chilean Government
2011	Aspectos bioéticos en el control y aplicación de plaguicidas en Chile	Acta Bioethica	Muñoz
2011	Estrategias globales para reducir el consumo de sal	Archivos Latinoamericanos De Nutrición	Valenzuela et al.
2011	El terremoto de 2010 en Chile: respuesta del sistema de salud y de la cooperación internacional	Revista Panamericana De Salud Pública	López et al.
2012	Ética de la Investigación: 10 años de experiencia docente.	Acta Bioethica	Moreno-Exebio
2012	Immigrant health workers in Chile: is there a Latin American ‘brain drain’?	Revista Panamericana De Salud Pública	Cabieses et al.
2012	Implementación del reglamento sanitario internacional (2005): reporte de progreso	El Vigia	Aguilera et al.
2012	La seguridad internacional en la proyección de Chile hacia el cono sur: ¿Desde la doctrina de la seguridad nacional hacia la construcción de comunidades de seguridad o la emergencia de la securitización?	Revista De Relaciones Internacionales, Estrategia Y Seguridad	Ovando et al.
2012	Neoliberalismo multicultural en el Chile postdictadura: la política indígena en salud y sus efectos en comunidades mapuches y atacameñas	Revista De Antropologia Chilena	Bolados et al.
2012	La investigación en salud: más allá́ de la ayuda internacional	Revista Médica De Chile	Solimano et al.
2013	América Latina frente a los determinantes sociales de la salud: Políticas públicas implementadas	Revista De Salud Pública	García-Ramírez et al.
2013	El derecho a la salud: su concepto en Chile y lo proclamado por la ONU	Medwave	Rosas
2013	Equidad en salud en la región más desigual del mundo: un reto de políticas públicas en América Latina	Revista Peruana De Medicina Experimental Y Salud Pública	Frenz et al.
2013	Changing patterns of migration in Latin America: how can research develop intelligence for public health?	Revista Panamericana De Salud Pública	Cabieses et al.
2013	Una mirada a la seguridad internacional a la luz de las estrategias de seguridad nacional	Estudios Internacionales	Dockendorff et al.
2014	Análisis biotecnológico de Latinoamérica a través de las patentes en Inmunología	Revista Cubana De Información Em Ciencias De La Salud	Campos et al.
2014	La dicotomía entre lo macroeconómico y el desarrollo humano	Estudios Internacionales	Riveros et al.
2014	Gobernar territorialidades transfronterizas: Seguridad y ‘desarrollo con identidad’ aymara en la triple frontera del norte de Chile (Chile-Perú-Bolivia)	Revista Trace	Rouvière
2014	Hacia una política integral para los trabajadores de la salud	Medwave	Becerra et al.

## Results

Public health scholars have only rarely engaged with IR theory or other relevant theoretical traditions when trying to understand GHD processes [6], with theoretical explanations tending to focus on the role of dominant states in pursuing security and economic interests, with little effort to examine the role of Southern partners in GHD. We draw on the field of IR and use of the concept of levels of analysis to organize our findings of drivers of health in Chilean foreign policy [7], distinguishing between: (i) the international/global level of analysis which focuses on the role of systemic factors as overarching policy constraints on foreign policy, including the distribution of power and norms in the international system, and the role of global actors (e.g. international non-governmental organizations (INGOs), international organizations (IOs), multi-national corporations (MNCs), and transnational policy advocacy networks in foreign policy priority setting; (ii) the domestic/state level of analysis which recognizes how differences in the political structure and culture of a nation-state and various domestic political actors (such as NGOs and economic interest groups) influence foreign policy formulation; and (iii) the individual level of analysis which emphasizes the role of individuals, especially key politicians and celebrities, and their perceptions and position in the foreign policy decision-making process [3]. For this review, the motivations behind efforts to advance GHD were categorized at three levels of analysis slightly different from the traditional IR axiology (international, regional and national) due to the prominence of regional health diplomacy, or SSC in health, across Latin America. We do not include individual drivers (i.e. celebrity diplomacy or personal policy champions) in the discussion as information on them was negligible in our literature sample.

### International/global drivers of GHD

The international/global is the dominant level of analysis in our article sample, being present in 21 of our sample total of 32 articles. Much of the writing on Chilean GHD focuses on the extent and quality of the Chilean state and non-state actors’ compliance with international health conventions, treaties, regulations, and standards that emanate from international organizations, mainly within the UN system [8–11]. Indicative of the international/regional orientation of much Latin American GHD, however, some studies compare Chilean policies and practices based on international conventions with reference to more developed countries [12, 13] or other countries in the Latin-American region [14–23].

The systemic driving forces of GHD invoked most frequently in the article sample is the pursuance of security at the global level, an instrumental framing in which health concerns are used strategically by nation-states to pursue security and other national goals within the international system, which some have called the securitization of health in foreign policy [24]. Several articles frame health as a national, as well as global, security issue, with specific reference in one study to Chile [25]. The authors of this study lay out the current debate between more restrictive and more inclusive definitions of security and suggest that while health issues are not included in narrower conceptualizations of national security, Chile has explicitly adopted a broad concept by including, among others, the following threats in its National Security Strategy: drug trafficking, natural disasters, and gun control [25]. In a similar vein, the United Nations, among other international organizations, has used definitions that include health risks, such as natural disasters and pandemics, in the concept of security. A frequently referenced aspect of global health security is Chile’s obligations under the WHO’s International Health Regulations (IHRs) (2005), and IHR requirements for changes at the Chilean national and regional level [8, 26].

A commonly described international driver of health issues in Chilean foreign policy are the mandates, regulations, and agreements originating from international organizations (IOs) to which Chile is either a formal signatory or an expressed adherent. Frequent reference in the literature is made to the Food Based Dietary Guidelines (FBDG) as a basis for ascertaining best practice options for Chile [13]; it is also worth noting that Chile’s 2017 legislation on front-of-pack food labelling for packaged foods high in fat, salt, sugar, and/or calories is considered a model for other Latin American countries to follow [27]. Another theme, invoking both global and regional analyses, is Chile’s response to WHO and PAHO strategies to ensure an adequate supply of human resources for health, proposing improved policy and practice in human resource management for the country [28]. While these cases indicate an active engagement in Chilean domestic policy in response to international guidelines, Chile was considered slow to take up the social model of health advanced by WHO health promotion declarations, only introducing a social determinants of health approach in 2007 [29]. The reasons for engaging actively with some, but not all, global policy innovations is likely political or due to constrained resources, but could weaken the standing of health in Chilean foreign policy over the longer term [2]. Chile’s docile adherence to some international agreements is consistent with its embrace of free trade agreements (FTAs), but regarded as inimical to health, also received critical commentary. This critique largely relates to intellectual property rights, i.e. the World Trade Organization’s Trade-Related Aspects of Intellectual Property Rights (TRIPS) agreement and subsequent free trade agreements with TRIPS+ provisions, and the resulting high costs of essential drugs, even as Chile refrained from taking full advantage of the flexibilization mechanisms regarding public health offered by the Doha Declaration on TRIPS and Public Health [30].

### Regional drivers of GHD

Regional cooperation has always played an important role in Latin American diplomacy, partly to counterbalance US historic dominance in the region. GHD activities are no exception to this general rule, with strong engagement by Chile on health diplomacy at the Union of South American Nations (UNASUR). With the creation of UNASUR in 2008, health policies became a strategic factor in Latin America to collectively balance the negative legacy of neoliberal policies in the region, with regional health diplomacy initiatives proliferating rapidly [31]. The Health Council of UNASUR is actively engaged in establishing health as a regional public good through the promotion of universal and equitable health systems across the region. It also aims to establish trans-border healthcare and access to the health services in each of the member countries for all inhabitants. This would represent an important achievement in a region with intense migration flows and where migrants often remain employed informally in host countries [31]; but it is also seen as an effort to build up an epidemiological shield.

Another central aspect of regional health diplomacy has been the coordination of emergency response in the aftermath of natural disasters. The UNASUR Health Council played an important role in coordinating the emergency response after the 2010 earthquake in Chile [31], while health worker migration has become a regional driver of GHD within Latin America. Most research on the phenomenon of ‘brain drain’ (i.e. the one-way flow of highly skilled/educated workers from one jurisdiction to another) has focused on movement from low- to high-income countries, with the significance of patterns of migration between middle-income countries such as those in Latin America being less clear. Although Chile itself is not experiencing significant international health worker migration (either inwards or outwards), studies on this phenomenon suggest distinctive patterns of migration within the Latin American region that deserve further research, with obvious implications for future regional health diplomacy [32].

A somewhat unique regional health diplomacy issue facing Chile is its shared borders with other countries having a high concentration of indigenous peoples. Convention 169 of the International Labour Organization (ILO) on indigenous peoples has significantly influenced new regional approaches, including Chilean efforts to construct a cross border political space for indigenous populations (primarily Aymaras) in Chile, Peru and Bolivia to reduce conflictual tensions between the three countries [23]. Pressure from both international and regional NGOs, in turn, has led Chile to initiate formation of a field of intercultural health aimed at incorporating indigenous healing practices into the public health system, while proposing pathways to the professionalization of indigenous health knowledge and practice for two indigenous groups in the Chilean territory [33].

### Domestic drivers of GHD

Pursuing economic interests in the context of globalization, linked to the hegemonic neoliberal ideology and dominance of global markets, constitutes a major driving force for GHD in Latin America, and especially in Chile. A common theme in the literature references the ongoing tension between the international drivers of foreign macroeconomic interventions in Latin America [34], including Chile’s adoption of neoliberal reforms during the 1970s and 1980s, and the regional drivers of self-consciousness amongst Chilean civil society informed by the problems such reforms brought to the country [21]. A key negative example of such international influences on Chilean health was the entrance of multinational financial capital in the health insurance and healthcare markets since 1981 and the influence foreign investors exercised on the direction of subsequent health sector reform [21]. In the last decades, there were attempts to shift the responsibility of access to care from individuals to the collective, but the results have been mixed. In terms of shifting power, regulations on the private sector were mainly ineffective while implemented reforms at the beginning of the century (a submarket of prioritized services with low copayments and totally funded services) partially “steered the market toward collective responsibility” [35].

As a major exporter of agricultural and livestock products, Chile favours protecting the integration of economic and health interests in its foreign policy, with the latter generally subsumed under and subordinate to the former. At times this has led to Chile being at variance with compliance of international health and environmental standards and regulations, notably concerning use of pesticides in agricultural, particularly fruit, production. Although Chile ratified the Stockholm Convention on Persistent Organic Pollutants (POPs) in 2004, application of this Convention in agricultural production has been challenged by the pesticide sales industry (multinational corporations), Chilean producers, and the Senate House of the Chilean Parliament [36]. Chile, similarly, has vacillated in its adherence to best practices of international pharmacovigilance in relation to veterinary medication products [37]. On a more positive note, Chile enthusiastically joined a sub-regional initiative in animal and human health in the Southern Cone Sub Regional Project on Cystic Echinococcosis (tapeworm) Control and Surveillance, an instance of neighboring countries reaching out to each other to solve a common economic and health problem [19].

The literature reviewed also displays a strong focus on the high degree of social inequity prevailing in Chile and the wider Latin American region, which directly impacts on access to healthcare and quality of life. This is reflective of the emerging global discourse about the detrimental impacts of growing social inequalities, including health inequities. The social approach to health, detailed in the final report of the WHO Commission on Social Determinants of Health [38], after 10 years has yet to produce concrete actions in Chile to achieve greater equality [16]. Despite one of the Commissioners being a former president of Chile, there are important political barriers to advance this approach. For example, the Chilean constitution, written during the dictatorship in the 1980s, only ensures the right to choose the type of health system (mostly public or private) that can be accessed, and not the more commonly constitutionally established “right to health” found in many other Latin American countries. Reducing the heavy weight of social inequalities in Chile, and Latin America more generally, is portrayed as the great pending task to a fuller implementation of the social determinants of health approach promoted by the WHO [18]. While some authors aknowledge that socio-economic development aimed at overcoming social inequities is important to achieve sustainable health gains for the whole population, most of them propose a combination of traditional health promotion interventions and gradual integration of the social determinants of health approach [11, 16, 21, 29, 39]. The Chilean experience in eradicating child malnutrition in the decades of the 60s through the 80s, through targeted, vertical, interventions (e.g. food supplementation), with no major changes in the country’s development process, is cited as such an example of ‘selective’ rather than comprehensive health intervention [29].

Another domestic driver of GHD is the occurrence of so-called ‘natural disasters’ (noting that these often arise, in part, from ‘unnatural’ human actions that, in turn, shape how ‘disastrous’ the consequences might be). This driver is unique to the Chilean context due to recurrent earthquakes, tsunamis, volcanic eruptions, floods, and forest fires. Chile has had to develop a set of strategies and policies to respond in the fastest and most effective ways possible to mitigate the damage, although studies have pointed out several weaknesses in the coordination of the international cooperation process, as well as the ‘lack of regulatory criteria for international cooperation in a situation of catastrophe…’ [40]. Health may be the incidental externality in such disasters, but the international dimension of relief efforts obviously invokes a certain degree of diplomacy.

A further domestic driving force for GHD is Chile’s pursuit of influence and leadership in the region. In achieving foreign policy goals, diplomatic efforts surrounding health are often understood in terms of the exercise of ‘soft power’ [41]. While hard power rests on the use of threats and violence, soft power seeks to obtain policy outcomes through leadership and cultural attraction without use of threats or sanctions. One soft power initiative is the 2008 Regional Ministerial Conference for the Eradication of Child Malnutrition in Latin America and the Caribbean, hosted by the Chilean Government, under the auspices of the first Bachelet Administration (2006–2010). The conference report has a strong focus on SSC and triangular cooperation (involving multiple partnering countries) and best practice sharing among countries of the region [42]. A related domestic driver for GHD is Chile’s search for improved relations with its neighbours (Argentina, Peru and Bolivia), a relationship that has been historically strained by border disputes. Chile’s bilateral relations with neighbouring Argentina, for example, has been peaceful and mutually beneficial since the signing of the Vatican mediated Peace Treaty of 1984, with both countries enjoying a sustained period of cooperation and integration in economic and social areas of common interest, including through health collaborations [43].

A more theoretical and less descriptive analysis goes further and suggests that the evolution of international cooperation in the Southern Cone region that began in the mid-1970s was informed primarily by the national security doctrine and, subsequently, by moderate constructivist approaches [44]. The latter approaches began receiving more attention as a way to address regional challenges, and is reflected in the use of concepts such as security communities, relational autonomy, and human security [45] that facilitated cooperation and integration of economic and social initiatives [22]. Chile is also frequently described as an actual or potential technical reference point for other countries of the Region, using its prestigious position in its international relations engagement [9, 12, 42]. Examples include presenting Chileans as ‘experts on public health events of International relevance…’ [8]; portraying Chile as having ‘…more solid institutional credibility’ than some of its neighbours; and that Chile can best ‘manage triangular cooperation… with the aim of teaching public management learned from other OECD (Organisation for Economic Co-operation and Development) members…’ [23]. These examples suggest that GHD activities present an opportunity for Chile to exert regional influence though its use of soft power.

## Discussion

While there is an emerging literature focused on explaining the driving forces behind the increasingly prominent role of health in foreign policy and global policy discussions [6, 46, 47], there have been few systematic attempts to theorize about what is driving this process. Existing scholarly interventions in this area are mostly advanced by IR scholars who study global health as a topical area of interest. This includes in the realist tradition how the role of security interests is driving global health, predominantly through narrow framing of health that prioritizes international security and economic concerns [48]; and from a constructivist perspective how health issue have become securitized [49], with a focus on the role of norms and values this process [50] and the application of principal-agent theory [51].

On the public health side, there are equally important but limited attempts to theoretically come to grips with GHD. For example, Kickbusch’s framing analysis, drawing on insights from macrosociology and health, explains how health is related to foreign policy, arguing that health has become a foreign policy issue due to its interlinkage with global security, economic and social justice agendas [46, 52] provide key determinants of effective GHD initiatives without theoretically grounding their explanatory model, while others [47] identify four main factors to explain successful GHD practice: political leadership; appropriate sequencing of the negotiation process, capacity-building; and an active role of non-governmental organizations, without linking this explanation theoretically to constructivist policy advocacy theories. However, existing conceptual contributions rarely systematically explain outcomes of GHD or theoretically relate those outcomes explicitly to key driving forces (actors, structures, institutions, etc.) [6]. Yet, this is exactly what is required for more theoretically rigorous explanations of GHD to emerge.

The theoretical underdevelopment of GHD is partly due to limited engagement of public health scholars with international relations theories, and the paucity of works of international relations scholar to, in turn, engage more systematically with health issues. This lack of cross-fertilization of knowledge has hampered efforts to develop better theoretically grounded understandings of GHD [3]. The discussion section will draw on four competing international relations theories to put forth a more theoretically grounded understanding of Chilean GHD and place our findings in a theoretical context: realism, liberalism, constructivism, and cosmopolitan (ethical) theory.^1^

Realists start from the assumption that foreign policy is predominantly driven by security interests in an anarchical international system in which states must guarantee their own survival [7]. A state’s position (of power) within the international system will dictate policy choices which are externally imposed by systemic constrains and international factors. From this perspective, the integration of health concerns into foreign policy discussions and negotiations is primarily driven by security interests, in particular the threat of rapidly spreading diseases linked to globalization.

Following the realist logic, Chile inherited, until recently, much of its foreign-policy dynamics from the national security doctrine, installed during the Cold War period and developed locally from the mid-1970s to the 1990s. This predominantly state-centric approach to international relations focusing on national security (and not broader conceptualizations of human security) is still present in part of Chilean foreign policies and is articulated and supported argumentatively within some of the reviewed articles. From the health perspective, this framework explains the adoption and construction of specific directives related to security, examples of which are discussions on the implementation of the International Health Regulations [8, 26], the development of pharmacovigilance systems [37], and the response to emergency and disasters [31, 40]. Although there is an effort to conceptualize multidimensional aspects of security, state-centered regulatory elements still predominate and the political narrative is centered on confronting security threats.

For liberal IR theory, the driving forces of foreign policy are domestically organized interest groups, especially economic interest groups, and GHD activities are mediated by economic and commercial interests [7]. Liberals contend that international factors are not able to fully account for the foreign policies of and interactions between countries, and that domestic political processes are central in driving foreign policy choices. Liberal IR theory further questions the notion that foreign policy is solely the purview of the state Executive.

Recent scholarship has questioned the traditionally dominant realist interpretation of Chilean foreign policy, and instead interrogated the domestic political process to identify the domestic driving forces of Chilean foreign policy adopting a more liberal IR approach [53]. This rise in the liberal approach to GHD, is arguably related to the growing importance placed on trade and economic development issues in Chile’s foreign policy. Its participation in international treaties and compliance with their terms, particularly of commercial treaties, are important in the construction of the country’s foreign policy, which persists beyond the time period developed for this review due to Chile being perceived as successful in its economic development over the past 25 years [12]. Only recently, and perhaps related to Chile’s participation the OECD, have other foreign policy concerns been raised within the country’s foreign policy establishment [54].

The foreign policy emphasis on economic factors has the potential to generate conflict with health needs, notably in Chilean compliance with intellectual property rights in trade agreements that create mandatory patents of drugs that compromises other commitments it has made to improve public health [30]. In the same way, at the domestic level, the heavy orientation towards economic development has worsened income inequalities, with impacts on access to and protection of health for certain population groups, such as people diagnosed with conditions not included in public prioritized strategies or uninsured groups. Although some of the articles present health as a global public good or as part of the right to health, there is no evidence that these concepts have been broadly implemented or accepted within Chilean politics. Rather, health and, by extension, global health, are considered part of the dynamics of healthcare as a market good, including the participation of multinational corporations such as private health insurers or care providers.

The interest- and security-centered understanding of GHD inherent to realist and liberal IR theories has been criticized as too narrow from both constructivist and cosmopolitan (or ethical) theoretical perspectives. Constructivists argue that actors’ interests expressed in the international domain are not materially given but are based upon their ideational understanding of the material structures surrounding them [55]. The very process of interactions through diplomatic channels can affect how states and non-state actors formulate their political preferences and articulate their interests. Thus health diplomatic processes become means by which states and non-state actors intersubjectively construct and express their ideas, interests, and identities [56], leading to reformulation of the very interests that cooperation was initially based upon.

The progressive constructivist vision of international relations developed in the aftermath of the Cold War has meant a rise globally in the status for health issues, with such issues being strongly influenced by international organizations and NGOs which have played a role in shaping GHD processes through policy advocacy. An example of this in Chile is the implementation of WHO health guidelines, such as the adoption of the perspective of social determinants of health and the adoption of WHO food guidelines and reduction of sodium consumption, eventually leading to Chile’s international leadership in processed food labelling requirements. This process, however, has had little impact on the construction of the domestic policy environment aimed at developing the health system to provide better health-sector responses to broader social determinants of health. Finally, constructivists highlight the importance of reputation and use of soft power in diplomatic encounters, with the exertion of regional influence based on reputation and expertise [41]. Chile is seen as a leader in cooperation policies within the region, such as confronting global epidemiological threats, control of infectious diseases, eradicating malnutrition, and implementing policies on childhood protection. Ideological leadership is also reflected in Chile’s entry into the OECD [57] and the recognition of the country as a hub for articulating triangular cooperation strategies.

The cosmopolitanism (ethical) perspective on the integration of health into foreign policy suggests that as a result of globalization’s putative erosion of border barriers and diffusion of digital technologies and the information revolution, existing health disparities between nations, such as differences in life expectancy and the under-five mortality rate, have become both readily and almost instantly observable. These increasingly apparent, and in some cases growing, health disparities have invoked a global response, and contributed to the growing notion of a single global community [58]. In other words, what is driving the integration of health into foreign policy are not security interests (at least not singularly) but a shared morality and duty to contribute to lasting health improvements amongst vulnerable populations globally. While cosmopolitan theory is least present in the literature we reviewed, some of the articles nevertheless draw on ethical arguments in their focus on health problems in specific populations, such as the professionalization of indigenous knowledge of native populations [33] or the urgent need for developing a better understanding of the relationship between migration and health [14].

Our discussion above indicates that the dominant explanations of GHD rooted in realist theory and focused on how the powerful agenda setters (predominantly the G7 and BRICs countries) promote security and economic interests through global health cooperation are still important but not fully able to explain SSC in the area of health. Instead, our review of Chilean GHD reveals a growing and complex mix of motivational factors behind the integration of health into foreign policy that not only responds to hegemonic perspectives. These motivations range from security and economic self-interest, to civil society advocacy, regional reputation building, and altruistic forms of health cooperation. Some have attempted to capture such multi-facetted motivations of Southern health cooperation through invoking the notion of soft power [41]. But such a view understands solidarity-oriented Southern health cooperation solely in instrumental terms, and denies the role of regional cooperation as a form of anti-imperialism rooted in historical experiences.

This is not to suggest that GHD in SSC is bereft of underlying security or economic self-interest. The best example for this is Brazil’s involvement in health cooperation with African countries. Brazil’s commitment to ‘structural health cooperation’ with African countries emphasizes capacity building in the health sector through unconditional investments in country-determined needs and involves long-term investments in health and social infrastructure relevant to the social determinants of health [59]; yet deep investments in mining and construction operate in parallel with health investments, guaranteeing access to much-needed resources and opening up new markets for Brazilian export products [4]. As such, health cooperation functions as an entry point to pursuing non-health related foreign policy objectives, but on a more equal footing than in traditional North-South cooperation and with more meaningful policy ownership by recipient countries.

This suggests that a different approach is needed when analyzing GHD in countries where SSC plays an important role (as compared to North South Cooperation). Due to the absence of significant asymmetries of power between countries in SSC, this framework should emphasize the importance of strategic cooperation of actors in geopolitical regions. It also needs to be open to incorporate a number of competing and contrasting explanations, such that it will be impossible to develop a single theory of GHD; rather we propose an eclectic approach to theorizing that takes into consideration that depending on the topic of interest (for example whether health issues have an inherent security dimension) multiple theories, styles, or ideas can gain complementary insights into GHD, with different theories arguably best applied in particular cases and at different levels of analysis (realism to understand security-driven GHD at the global level; liberalism for economic interest-driven GHD at domestic level; and constructivism to understand regional and normatively driven cooperation in GHD).

### Limitations and strength of the review

This review included only published documents on a limited set of databases, and thus omitted results from non-published (or non-indexed) works. Through the inclusion of only Spanish and English language documents we might have missed some results published in Portuguese, a language that produces good quality evidence from Brazil. As is common when conducting a narrative literature review, we did not assess the quality of the articles included in the review. Consequently, any conclusion is “open to bias from the potential to omit, perhaps inadvertently, significant sections of the literature or by not questioning the validity of statements made” [5]. We also did not account specifically for the different types of articles reviewed (except by the kind of publication). However, this opening up of the inclusion process made it possible to present a broader perspective on the emerging policy issue surrounding Chilean GHD [60].

## Conclusion

Even though there is a paucity of studies, our literature analysis reveals some significant insights regarding the evolving application of the concept of Global Health Diplomacy in Chile. The relevance of international institutions such as the WHO and its cooperating regional organization (PAHO) is present either as a driver for some specific initiatives in Chile or as a coordinating agency for international cooperation at the regional level. There is incipient awareness about the importance of assessing the impact of international trade on the human right to health and wellbeing, rather than simply assuming its superordinate importance. Moreover, the paradigm of social determinants of health seems to have gained some ground, including an understanding of health inequities as an aspect of global phenomena and not just of local conditions. As in most emerging research fields, and specifically in a country that has not yet entered a stage of development that correlates strongly with equitable improvements in socioeconomic status, more studies are needed in global health, specifically as it relates to diplomacy.

In light of our analysis, and from a theoretical point of view, health actions in the context of international relations in Chile are still mainly motivated by more traditional foreign policy interests rather than by a desire to satisfy health needs per se, that is, health is a means to foreign policy goals rather than the inverse [61]. This is especially apparent at the global level where security interest remains at the heart of many GHD activities. This seems to conform with findings of existing GHD scholarship that emphasize the importance of security and economic interests as driving forces of GHD, and how health is often appropriated instrumentally within foreign policy settings to achieve other goals.

But the literature reviewed also shows that in Chile there has been an evolution from chiefly domestically focused health policies (e.g. maternal and child nutrition treatment) towards internationally inspired integrated policies (e.g. maternal and child nutrition promotion aligned with international guidelines). This evolution has led to the application of multidisciplinary and intersectoral approaches in the development of Chile’s foreign policies. Health concerns are gradually appearing in the country’s international relations agenda, initially through the adoption and enactment of UN and other international agencies’ mandates, conventions, and guidelines. But security and economic interests are often still underpinning or mediating those foreign policy interests (e.g. health security), while exertion of soft power, reputation building, and regional solidarity can also drive the foreign policy agenda.

We therefore conclude that no single IR theory discussed in this article can fully explain, or provide a conceptual foundation for, all health phenomena, relations, and interests at play in Chile’s global political processes. Instead, theoretically grounded explanations of GHD should focus on how different theoretical elements can be useful under different conditions and at different levels of analysis, for example: realism by explaining security driven global health cooperation at the global level; constructivism by explaining normatively driven health cooperation at the regional level; and liberalism for explaining how health cooperation can be driven by economic (and other) interest groups domestically. In essence, efforts to theorize must recognize how different aspects of various IR theories, as conceptual lenses, will provide complementary insights into developing a better understanding of GHD, without a single theory able to capture the complex practices that encompass GHD [62].

In addition, original analyses not widely used in IR (such as complexity theory, systems theory, and network mapping) could further complement IR theories to add more nuance to an understanding of the interplay in GHD between hard power, soft power, domestic (liberal) interest, and normative (constructivist) or ethical forces at play in defining how countries such as Chile come to position health as a foreign policy goal. Yet it remains clear that the historical experiences of colonialism and imperialism have shaped the perceptions and practices of GHD amongst many Southern countries, including Chile, which increasingly see GHD as an opportunity to pursue more egalitarian forms of cooperation that delink health cooperation from traditional foreign policy and commercial objectives, even as those remain lurking in the background.

## Additional files

Additional file 1: Flow Diagram. Contains details of the broad search strategy used in this paper. (DOCX 115 kb)

Additional file 2: Search terms. Contains a list of concepts and terms used in this review. (DOCX 58 kb)

Additional file 3: Codes. Contains a list of Dimensions and names of the codes used to classify information from the articles, and the number of articles that included each code. (DOCX 57 kb)

## Abbreviations

ASCE: American Society of Civil Engineers
BIREME: *Centro Latinoamericano y del Caribe de Información en Ciencias de la Salud* (Latinamerican and the Caribean Center of Health Sciences Information)
BRICS: Brazil, Russia, India, China and South Africa
CAN: *Comunidad de Naciones Andinas* (Andean Community of Nations)
DSG: *Diplomacia en Salud Global* (Global Health Diplomacy)
FBDG: Food Based Dietary Guidelines
FTAs: Free Trade Agreements
G7: Group of Seven (Canada, France, Germany, Italy, Japan, the United Kingdom, and the United States)
GHD: Global Health Diplomacy
H1N1: Influenza A (H1N1)
IHRs: International Health Regulations
ILO: International Labour Organization
INGOs: International Non-Governmental Organizations
INIA: *Instituto Nacional de Investigaciones Agropecuarias* (National Institute of Agricultural Research)
IOs: International Organizations
IR: International Relationships
MERCOSUR: *Mercado Común del Sur* (Common Market of the South)
MINREL: *Ministerio de Relaciones Exteriores* (Foreign Affairs Ministry)
MNCs: Multi-National Corporations
NGOs: Non-Governmental Organizations
OECD: Organization for Economic Co-operation and Development
PAHO: Pan-American Health Office
POPs: Persistent Organic Pollutants
SARS: Severe acute respiratory syndrome
SSC: South-South Cooperation
TRIPs: Trade-Related Aspects of Intellectual Property Rights
UN: United Nations
UNASUR: *Unión de Naciones Suramericana* (Union of South Americans Nations)
US: United States
WHO: World Health Organization
WTO: World Trade Organization

## Footnotes

1 For the purpose of this article, we will not distinguish between different sub-strands of IR theories. However, we do acknowledge that, for example, within realism there are several sub-strands with significant differences in how each understands foreign policy choices (e.g. classical realism [based on theories of Hans Morgenthau and Reinhold Niebuhr] which emphasizes the role of individuals, structural (or neo-) realism which privileges international systemic factors [initiated by ideas of Kenneth Waltz], and neoclassical realism [concept developed by Gideon Rose] which opens up the black box of the state to domestic analysis).
