# Supplementary material for: Chile’s role in global health diplomacy: a narrative literature review
Source: Global Health. 2018 Nov 16;14:108. doi: 10.1186/s12992-018-0428-8 (PMC6240220; doi:10.1186/s12992-018-0428-8)
Supplement: Supplementary file 1 — Flow Diagram. Contains details of the broad search strategy used in this paper. (DOCX 115 kb) [file 12992_2018_428_MOESM1_ESM.docx]

**Appendix A**

**Flow Diagram**

**Inclusion criteria**

Articles in the Chilean context that:

- Cover international policy issues relevant to health due to their direct and / or indirect implications.
- Discuss (explicitly or implicitly) how health issues are framed / incorporated in foreign policy negotiations.
- Analyze the forces involved in the integration of health in foreign policy negotiations
- Explore the ways in which diplomacy in global health is practiced.
- Analyze explicitly and / or implicitly the bases or theoretical foundations of the integration of health in international politics, in terms of international relations theory.

**Exclusion criteria**

Articles published before 2000.

Articles not in Spanish or English.

Title Screening: 177

Abstract Screening: 78
