# Supplementary material for: Chile’s role in global health diplomacy: a narrative literature review
Source: Global Health. 2018 Nov 16;14:108. doi: 10.1186/s12992-018-0428-8 (PMC6240220; doi:10.1186/s12992-018-0428-8)
Supplement: Supplementary file 2 — Search terms. Contains a list of concepts and terms used in this review. (DOCX 58 kb) [file 12992_2018_428_MOESM2_ESM.docx]

Appendix B

Search terms

| **AND** | | | | |
| --- | --- | --- | --- | --- |
|  | **Concept 1** | **Concept 2** | **Concept 3** | **Concept 4** |
|  | **Relaciones internacionales** | **Diplomacia**  **Internacional** | **Salud** | **Chile*** |
| **OR** | Derechos humanos | Diplomátic* | Sanitari* | Sudamérica |
|  | Negociaciones internacionales | Económic* | Seguridad | América del Sur |
|  | Propiedad intelectual | Economía | Enfermedad | América latina |
|  | Tratado | Política exterior | Epidemia* | Latinoamérica |
|  | Acuerdo | Global | Epidémic* |  |
|  | Desarrollo | Canciller* | Pandémic* |  |
|  | Reglamento | Bilateral* | Pandemia* |  |
|  | Gobernancia | Multilateral* |  |  |
|  | Gobernanza |  |  |  |
|  | Equidad |  |  |  |
|  | ODM |  |  |  |
|  | Organismo |  |  |  |
|  | Organizaciones |  |  |  |
|  | Organización |  |  |  |
|  | UNITAID |  |  |  |
