# Supplementary material for: Chile’s role in global health diplomacy: a narrative literature review
Source: Global Health. 2018 Nov 16;14:108. doi: 10.1186/s12992-018-0428-8 (PMC6240220; doi:10.1186/s12992-018-0428-8)
Supplement: Supplementary file 3 — Codes. Contains a list of Dimensions and names of the codes used to classify information from the articles, and the number of articles that included each code. (DOCX 57 kb) [file 12992_2018_428_MOESM3_ESM.docx]

Appendix C

Codes

| **Dimension** | **Name of the Code** | **Code** | **Nº of articles including the topic** |
| --- | --- | --- | --- |
| **Definitions** | Global Health | DSG | 3 |
|  | Diplomacy in Global Health | DDSG | 3 |
| **Drivers** | Regional / Domestic | DREG | 13 |
|  | International | DINT | 21 |
| **Framing of Health** | Development | MD | 10 |
|  | Ethical issue | MTE | 7 |
|  | Global Public Good | MBPG | 3 |
|  | Human Right | MDDHH | 3 |
|  | Security | MSEG | 8 |
|  | Trade | MCOM | 5 |
| **Process** | GHD Process | PRO | 11 |
| **Policy Choices** | Health in all Policies | OPST | 8 |
|  | Health Impact Assessment | OPIMPA | 5 |
|  | Soft Power Mechanisms | OPPRES | 5 |
|  | Diplomatic Training | OPENT | 1 |
|  | Other policies | OPOP | 12 |
| **Data** | Empirical Data | DEMP | 15 |
| **Methodology** | Methodology | METO | 12 |
| **Others** | Others SDS | ODDSS | 3 |
|  | Others | OTRO | 33 |
